# Perioperative fluid dynamics evaluated by bioelectrical impedance analysis predict infectious surgical complications after esophagectomy

**DOI:** 10.1186/s12893-019-0652-z

**Published:** 2019-12-02

**Authors:** Shuichiro Oya, Hiroharu Yamashita, Ryohei Iwata, Koichiro Kawasaki, Asami Tanabe, Koichi Yagi, Susumu Aikou, Yasuyuki Seto

**Affiliations:** 1 Department of Gastrointestinal Surgery, The University of Tokyo, 7-3-1, Hongo, Bunkyo-ku, Tokyo, 113-8655 Japan; 2 Department of Bariatric & Metabolic Care, Graduate School of Medicine, The University of Tokyo, 7-3-1, Hongo, Bunkyo-ku, Tokyo, 113-8655 Japan

**Keywords:** Esophagectomy, Body fluid balance, Bioimpedance analysis, Postoperative complications, Infectious complications

## Abstract

**Background:**

Transthoracic esophagectomy, among the most invasive surgeries, is highly associated with postoperative infectious complications which adversely affect postoperative management including fluid dynamics. The aim of the study is to evaluate the utility of perioperative bioelectrical impedance analysis **(**BIA) measurements for the patients after transthoracic esophagectomy.

**Method:**

Multi-frequency BIA measurements were conducted in 24 patients undergoing transthoracic esophagectomy preoperatively, at 1 h after surgery, and twice daily for the following 7 days. The amounts of extracellular water (ECW), internal cellular water (ICW), total body water (TBW), and fat-free mass (FFM) were calculated. Changing trends in variables were analyzed, and the patients were subdivided according to the presence of infectious surgical adverse events to identify differences in fluid dynamics.

**Results:**

ECW was the major body fluid compartment showing an increase after surgery, and peaked on postoperative day (POD) 2. Twelve patients experienced infectious complications. The peaks of changes in ECW and ECW/TBW appeared earlier and their values at the highest peak were significantly lower in the group without infectious complications on POD 2. The ICW/FFM value showed a mild decrease as compared to POD1 and then gradually recovered. It was significantly lower even before surgery and showed the most significant stratification on POD2. ECW/TBW of 48% and ICW/FFM of 37% on POD2 were predictive cut-off values for infectious adverse events with high area-under receiver operating characteristic (ROC) curves: 0.80 or higher.

**Conclusion:**

BIA measurements are useful for monitoring fluid retention and may predict infectious complications in the early phase after transthoracic esophagectomy.

**Trial registration:**

Registry name: UMIN-CTR, ID: UMIN000030734, Registered on January 9, 2018, retrospectively registered.

## Background

Understanding the fluid dynamics modulated by surgical stress is essential to managing perioperative patients. Body fluids begin to shift, after a surgical intervention, from the intravascular to the interstitial compartment. Thereby, these fluids do not contribute to blood circulation due to destruction of the endothelial glycocalyx, a key vascular barrier structure [1]. As the patient recovers from surgical stress, the body fluids in the interstitial compartment begin to redistribute into the intravascular compartment, i.e. fluid retention manifests. This postoperative event generally occurs within a few days and the extent of fluid dynamic changes reflects the invasiveness of the surgical procedure. Therefore, it is mandatory, especially in patients who have undergone invasive surgery, to carefully manage the infusion volume especially during the fluid redistribution phase. Preventing fluid overload as well as the resultant burden on the respiratory and cardiovascular systems is essential to avoiding a variety of postoperative adverse events.

Patients with systemic inflammatory responses secondary to infectious complications usually require more fluid administration to maintain adequate circulation during the postoperative period. The circulation is partially supported by immediate fluid resuscitation, which should reportedly be carried out within the first three hours to prevent sepsis-induced hypo-perfusion [2]. Therefore, the extent of fluid administration and subsequent retention is potentially greater in post-surgery patients with infectious complications, even before they manifest clinically, potentially necessitating even more meticulous management thereafter.

Transthoracic esophagectomy is one of the most invasive surgeries and is associated with high rates of morbidities including pneumonia, anastomotic leakage, and surgical site infection [3]. Given that this procedure is associated with not only high invasiveness but also a high incidence of infectious complications, postoperative fluid management is often a challenging issue in this population. Consequently, fluid restriction, which is more likely to achieve good outcomes than less strict fluid management strategies [4, 5], is not widely employed.

As a novel tool for evaluating body fluids, BIA measurements have been attracting considerable attention in the fields of nutritional assessment [6, 7] and critical care [8, 9]. BIA measurements can be employed for perioperative fluid management as well, and the usefulness of this approach has been reported [10–15]. This study aimed to assess the trends in perioperative fluid distribution changes, by employing multi-frequency BIA measurements. We also evaluated possible differences according to the presence of infectious adverse events in patients who had undergone transthoracic esophagectomy.

## Patients and methods

### Patients

Twenty-five patients with esophageal cancer undergoing transthoracic esophagectomy between June 2017 and April 2018 participated in this study. One patient was excluded from the analysis due to conversion to a two-staged procedure intraoperatively. Intraoperative body fluid management and anesthesia consisting of inhaled general anesthesia and epidural anesthesia depended on the preference of the anesthesiologist. The overall goal of postoperative body fluid management was to maintain the mean blood pressure above 65 mmHg mainly by administering crystalloids, and albumin solutions and/or circulating agonists when the mean blood pressure did not respond to sufficient crystalloid administration. Red blood cell (RBC) transfusions were considered when the patient’s hemoglobin levels were lower than 8.0 g/dL at the perioperative periods.

Pathological staging was based on the Union for International Cancer Control (UICC) TNM staging system 8th edition for esophageal cancer [ 16]. Physical status before surgery was classified according to the system of the American Society of Anesthesiologists [17]; ASA-PS. Nutritional status before surgery was also evaluated using Prognostic Nutritional Index (PNI) by Onodera et al. [18] because of the possibility of influencing the postoperative course [19]. Postoperative complications were assessed using the Clavien-Dindo classification (C-D classification) [20]. We employed a prospective observational study design, at a single center, and obtained approval from the ethics committee of the Faculty of Medicine, The University of Tokyo.

### Recording body weights and cumulative fluid balances

Body weights and the cumulative fluid balances of the patients were recorded as the reference indicators in this study. Body weights were recorded once daily from the 1st to the 7th postoperative day (POD). Cumulative fluid balances (CFB) were calculated as the difference between the total fluid intakes and the outputs divided by the preoperative body weights. Insensible perspirations were not taken into account. The CFBs were calculated directly after the surgery and once daily at 12:00 from POD 1 to 7.

### BIA measurements

The BIA measurements were performed using a multi-frequency bioelectrical impedance analysis system (MLT-550 N, SK Medical Electronics Co., Ltd., Tokyo, Japan). BIA uses a low alternating electric current that does not affect the subject unless embedded devices, such as cardiac pacemakers, are present. The alternating electric current with low frequency tends to flow only through the extracellular fluid compartment, while the high frequency current flows to both the extracellular and the internal cellular fluid compartments. The resistance values which reflect opposition to the current flow through intra- and extracellular fluids, and the reactance values which reflect the capacity produced by interfaces of tissues and cell membranes [6], are both measured using these alternating electric currents with multiple frequencies. From the obtained resistance and reactance values and the subject’s height and other general information, the amounts of extracellular and internal cellular fluids contained in the subject’s body are calculated.

The patients were instructed to remain in the supine position with their upper limbs not touching their trunk and with the thighs also kept apart. Two electrodes were attached to the back of the right wrist and another two on the right foot. The measurements were carried out using alternating currents up to 250 micro amps with multiple frequencies ranging from 2.5 kHz to 350 kHz. The amounts of extracellular water (ECW), internal cellular water (ICW) and total body water (TBW) were calculated from the obtained resistance value, reactance value, sex and height information. The body weight was additionally required to calculate fat-free mass (FFM). The body weight one hour after surgery was estimated from the preoperative body weight and the cumulative fluid balance during surgery. When the body weight value was not available at other measurement time-points, the weight just prior to the measurement was applied for the FFM calculations. These estimated body weight values were not used for analyzing postoperative body weight trends.

The baseline BIA was measured two days before surgery and one hour after surgery, followed by two daily measurements in the morning (around 10:00) and evening (around 18:00) from POD 1 to 7. It was not recorded when the patient was under management with a life support device other than a mechanical ventilator or a hemodialysis device, nor when the patient refused the measurement due to physical discomfort after surgery or for any other reason.

### Estimations of fluid retention timings

The onset of initial fluid retention was defined as the first day of two consecutive days of weight loss after surgery, as conventionally estimated. Weight loss timing was compared with the peak change in each parameter obtained by BIA measurements in each patient.

### Definition of infectious complications

Major postoperative adverse events were recorded after the surgery and we defined postoperative pneumonias, anastomotic leakages, and other surgical site infections of C-D grade 2 or higher as the infectious complications. Diagnostic criteria for pneumonias included pulmonary infiltrative shadows on the chest X-rays or Computerized tomographic scannings (CTs), purulent sputum, and increased white blood cell (WBC) counts or C-related protein (CRP) levels. Anastomotic leakages and other surgical site infections were confirmed by CT or contrast agent test when both of the purulent effluent from drainage tubes and the elevation in WBC counts or CRP levels were observed. We divided the patients into two groups based on the occurrence of these infectious adverse events in an effort to identify differences between the groups in postoperative BIA parameter trend changes. The changing trends of the body weights and the CFBs were also compared between these groups.

### Statistical analysis

Statistical analyses were all performed with JMP Pro 13.2 software (SAS Institute Inc. Cary, NC, USA). Continuous variables were compared between groups by the Man-Whitney U test. In the logistic analyses, Fisher’s exact tests were used for categorical variables. Cut-off values for the receiver operating characteristics (ROC) curves were determined by Youden index. For the multivariate analyses, linear regression analyses using least-squares methods and multivariate logistic regressions with forced entry methods were carried out. *P* values less than 0.05 were considered to indicate a statistically significant difference.

## Results

### Changing trends in TBW, ECW and ICW

**Fig. 1:**
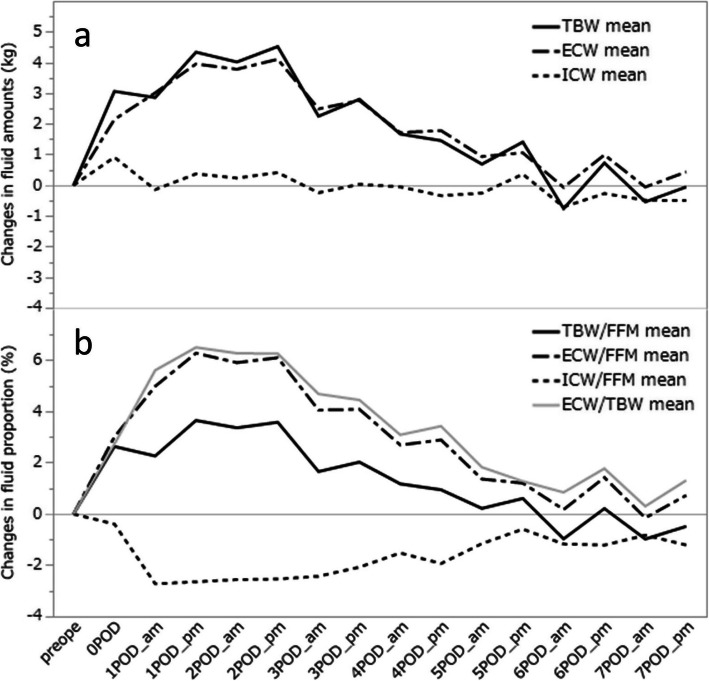
Changes in body fluid amounts (**a**) and changes in fluid proportions in patients after transthoracic esophagectomy (**b**). Increments from the preoperative state are shown for one week after surgery. TBW, total body water; ECW, extracellular water; ICW, internal cellular water; FFM, fat free mass

In total, 372 BIA measurements were performed in the 24 patients. Figure 1a shows the mean amounts of change in TBW, ECW and ICW from the preoperative levels (TBW-C, ECW-C and ICW-C) in our 24 patients. The mean ECW-C peaked on POD 1 to 2, whereas the mean ICW-C remained at approximately the preoperative level throughout the measurement period. Thus, the mean TBW-C, calculated as the sum of ECW-C and ICW-C, also peaked on POD1 to 2.

### Changing trends in TBW, ECW and ICW relative to FFM

Figure 1b shows the changes in the proportions of each body water compartment to FFM: ECW/FFM-C, ICW/FFM-C and TBW/FFM-C. The ECW/TBW (ECW/TBW-C) change is also shown. The mean ECW/FFM-C and TBW/FFM-C peaked, while the mean ICW/FFM-C reached the lowest value on POD1 to 2. TBW/FFM-C was lower than ECW/FFM-C and the mean ECW/TBW-C was equivalent to the mean ECW/FFM-C throughout the measurement period.

### Peaks of BIA parameters and their relationships with body fluid retention

The times of fluid retention were not defined in three cases since the body weight trend patterns did not meet the criteria. In most of the remaining 21 patients, the times of the maximum values in ECW, ECW/FFM and ECW/TBW appeared on the same day, or 1–2 days prior to the day that fluid retention manifested, while appearing a few days later in the others (Additional file 1: Figure S1). Correspondingly, the ICW/FFM minimum peaks appeared within two days before and after the day that fluid retention became apparent.

### Postoperative adverse events and their relationships with clinical factors

Table 1 shows the numbers of patients who experienced major postoperative adverse events and their incidence rates. Incidence rate of the pneumonias, leakages and other surgical site infections of C-D grade 2 or more were 37.5, 12.5 and 12.5%, respectively. These infectious complications developed in 12 patients including 4 who experienced multiple adverse events, and they were observed on POD3 or later except in three patients with pneumonia which developed on POD2. Two cases of acute respiratory distress syndrome (ARDS) were all accompanying other infectious complications.

**Table 1:** Incidence rates of postoperative adverse events observed in patients after open esophagectomy

Total		*n* = 24	(%)
Pneumonia	Grade2	6	25.0%
	> = Grade3a	3	12.5%
	Any	9	*37.5%*
ARDS	Any	2	8.3%
Leakage	Grade2	1	4.2%
	> = Grade3a	2	8.3%
	Any	3	*12.5%*
SSI	Wound	1	4.2%
	Mediastinitis	1	4.2%
	Empyema	1	4.2%
	Any	3	*12.5%*
Chylothorax	Grade2	1	4.2%
	> = Grade3a	1	4.2%
	Any	2	8.3%
Pleural fluid	> = Grade3a	6	25.0%
Pneumothorax	> = Grade3a	1	4.2%
Laryngeal N paralysis	> = Grade3a	1	4.2%
Delayed Emptying	> = Grade2	3	12.5%
Af	Any	9	37.5%

Infection-related events are shaded in the table. Severities of the adverse events are graded according to the Clavien-Dindo classification. Some cases had experienced overlapping episodes. *ARDS* Acute Respiratory Distress Syndrome, *SSI* surgical site infection, *Laryngeal N* laryngeal nerve, *Af* Atrial fibrillation

The patients were then divided into an infection group (*n* = 12) and a non-infection group (*n* = 12) in accordance with their complication occurrence status (Table 2). None of the factors listed differed significantly between the two groups.

**Table 2:** Comparison of patient characteristics with or without the infectious postoperative complications after transthoracic esophagectomy

		All (*n* = 24)	Infectious AE, > = Grade2		*P* value
			yes (*n* = 12)	no (*n* = 12)	
Sex	Male / Female	17/7	10/2	7/5	0.3707
Age	Mean ± SD	68.1 ± 7.7	70.5 ± 8.8	65.8 ± 6.4	0.1698
Height (cm)	Mean ± SD	161.9 ± 9.1	163.0 ± 8.6	160.8 ± 10.3	0.7439
Preoperative BW (kg)	Mean ± SD	56.5 ± 12.5	55.1 ± 13.6	58.0 ± 11.8	0.4010
BMI (kg/m^2^)	Mean ± SD	21.3 ± 3.4	20.6 ± 4.0	22.0 ± 2.7	0.4862
Preoperative therapy	none	5	3	2	1.0000
	Chemo/CRT	14/5	6/3	8/2	
ASA-PS	<=1 / > = 2	7/17	4/8	3/9	1.0000
PNI (Onodera et al.)	Mean ± SD	44.6 ± 5.7	44.5 ± 6.0	44.8 ± 5.8	0.9323
Reconstruction route	ITH / PMD / RST	10/10/4	6/5/1	4/5/3	0.6634
Lymph node dissection	<=2 / 3fields	12/12	7/5	5/7	0.6843
Operative time (min)	Mean ± SD	403.3 ± 54.7	394.8 ± 46.6	411.8 ± 64.8	0.3259
Intraoperative Blood Loss (mL)	Mean ± SD	513.3 ± 271.4	520.4 ± 285.9	506.3 ± 268.6	0.9081
RBC transfusion intraoperative early postope, ~2POD	No/Yes	23/1	11/1	12/0	1.0000
	No/Yes	17/7	8/4	9/3	1.0000
Steroid use (~7POD)	No/Yes	17/7	7/5	10/2	0.3707
Sivelestat use (~7POD)	No/Yes	16/8	6/6	10/2	0.1930
Resection	R0,1/R2	20/4	11/1	9/3	0.5901
pT	0/1a/1b/2	1/2/2/4	1/1/0/1	0/1/2/3	0.4003
	3/4	12/3	8/1	4/2	
pN	0	4	2	2	1.0000
	1/2/3	14/2/4	6/2/2	8/0/2	
pM^a^	0 / 1	23/1	12 / 0	11 / 1	1.0000

No patient, surgical or pathological factors, related to the complication incidence, were identified. pM^a^ includes metastasis involving the supraclavicular lymph-nodes. *AE* Adverse Events, *BW* body weight, *BMI* body mass index, *PNI* Prognostic Nutritional Index (preoperatively calculated), *ASA-PS* Physical state classification by the American Society of Anesthesiologists, *ITH* intrathoracic route, *PMD* post-mediastinal route, *RST* retrosternal route, *RBC* Red Blood Cell, *postope* postoperative, *pT/N/M* pathological T/N/M

### Relationship between infection and change in body weight, cumulative fluid balance

**Fig. 2:**
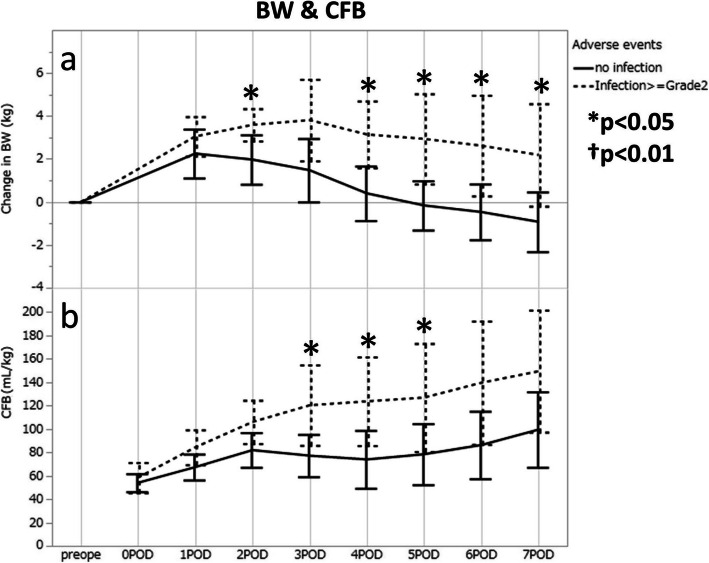
Changes in body weight increments (**a**) and the cumulative fluid balances (**b**) after open esophagectomy in patients, with and without infectious complications. Insensible perspirations were not taken into account for the CFB calculations. The mean values and the 95% confidential intervals are presented to show the trends within the same groups. BW, body weight; CFB, cumulative fluid balance. *p < 0.05, †*p* = 0.01 by Mann-Whitney U test

The trends of body weight changes from the preoperative levels are shown in Fig. 2a. Body weights peaked on POD1 in the non-infection group whereas the peak was on POD3 in the infection group, and the increment in the infection group was also higher than that of the non-infection group. The median value (25th percentile, 75th percentile) of Body weight changes was significantly higher in the infection group than in the non-infection group on POD2 [+ 3.19 kg (+ 2.81, + 4.26) vs + 1.75 kg (0.75, 3.48), *p* = 0.0376] and POD4 [+ 2.43 kg (+ 1.08, + 5.70) vs + 0.33 kg (− 1.02, 2.39), *p* = 0.0282] or later*.*

CFBs were also higher in the infection group throughout the week after the surgery (Fig. 2b), and the values were significantly higher in the infection group than the non- infection group on POD3 [+ 113.9 mL/kg (+ 72.4, + 157.2) vs + 70.9 mL/kg (+ 51.9, + 110.2), *p* = 0.0327], on POD4 [+ 117.6 mL/kg (+ 87.4, + 169.7) vs + 77.2 mL/kg (+ 39.1, + 102.0), *p* = 0.0284], and on POD5 [+ 124.4 mL/kg (+ 88.5, + 158.8) vs + 89.9 mL/kg(+ 37.3, + 114.4), *p* = 0.0449].

### Relationship between infection and change in ECW amount

**Fig. 3:**
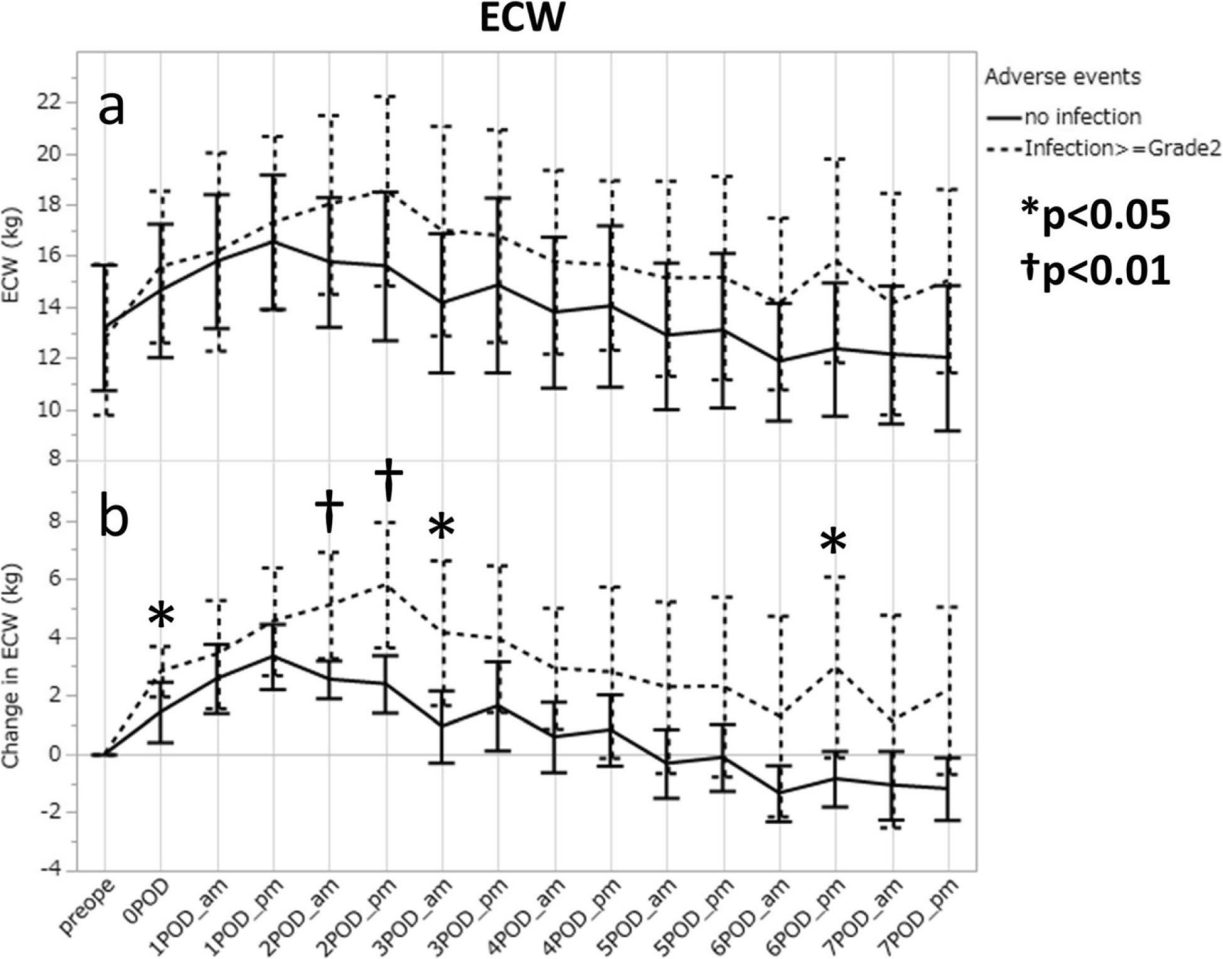
Changes in extracellular water amount after open esophagectomy in patients, with and without infectious complications; actual values (**a**) and increments (**b**). The mean values and the 95% confidential intervals are presented to show the trends within the same groups. ECW, extracellular water. *p < 0.05, †*p* = 0.01 by Mann-Whitney U test

The measured amount of ECW peaked in the evening of POD1 in the non-infection group and in the evening of POD2 in the infection group (Fig. 3a). In addition, the increment in the peak from the preoperative level was greater in the infection group (Fig. 3b). The median value (25th percentile, 75th percentile) of ECW-C was significantly higher in the infection group than in the non-infection group in the morning of POD2 [+ 3.90 kg (+ 3.40, + 7.80) vs + 2.40 kg (+ 1.75, + 3.45), *p* = 0.0081], in the evening of POD2 [+ 5.15 kg (+ 3.98, + 8.35) vs + 2.15 kg (+ 0.83, + 3.95), *p* = 0.0042], and in the morning of POD3 [+ 3.20 kg (+ 1.70, + 5.20) vs + 1.05 kg (− 0.38, + 2.60), *p* = 0.0209].

Considering the possible predictors listed on Table 2 except for the pathological status of the resected tumor, linear regression analyses for the ECW-C values on POD2 revealed that the patients who received intraoperative RBC transfusion and the patients without any neoadjuvant therapy tended to have higher ECW-C values in the morning of POD2 (*p* = 0.0220 and *p* = 0.0287, respectively), and the elderly patients also had higher ECW-C values in the evening of POD2 (*p* = 0.0337).

### Relationship between infection and the ICW/FFM value

**Fig. 4:**
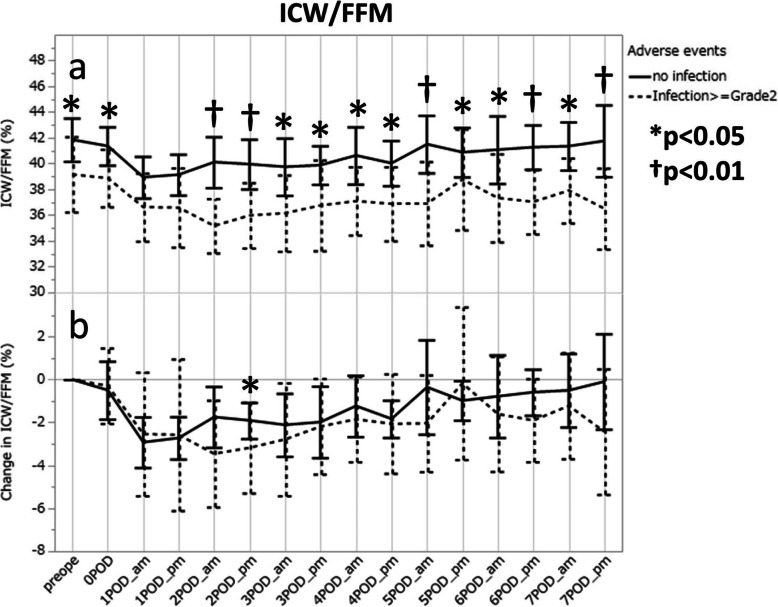
Changes in ICW/FFM after open esophagectomy in patients, with and without infectious complications; actual values (**a**) and increments (**b**). The mean values and the 95% confidential intervals are presented to show the trends within the same groups. ICW, internal cellular water; FFM, fat-free mass. *p < 0.05, †*p* = 0.01 by Mann-Whitney U test

The median value (25th percentile, 75th percentile) of ICW/FFM was significantly lower in the infection group throughout the BIA measurement period, even before surgery, but most remarkably in the morning of POD2 [35.5% (34.2, 36.2) vs 38.9% (38.2, 41.8), *p* = 0.0004] (Fig. 4a). In contrast, the ICW/FFM-C values, for the most part, did not differ between the groups during the measurement period except in the evening of POD2 (Fig. 4b).

Applying linear regression analyses for the ICW/FFM values on POD2 as the same way as for the ECW-C values, we found that female patients had higher ICW/FFM values than male patients only in the evening of POD2 (*p* = 0.0489). No other factors were relevant to the ICW/FFM values observed at any time points.

### Relationship between infection and the ECW/TBW value

**Fig. 5:**
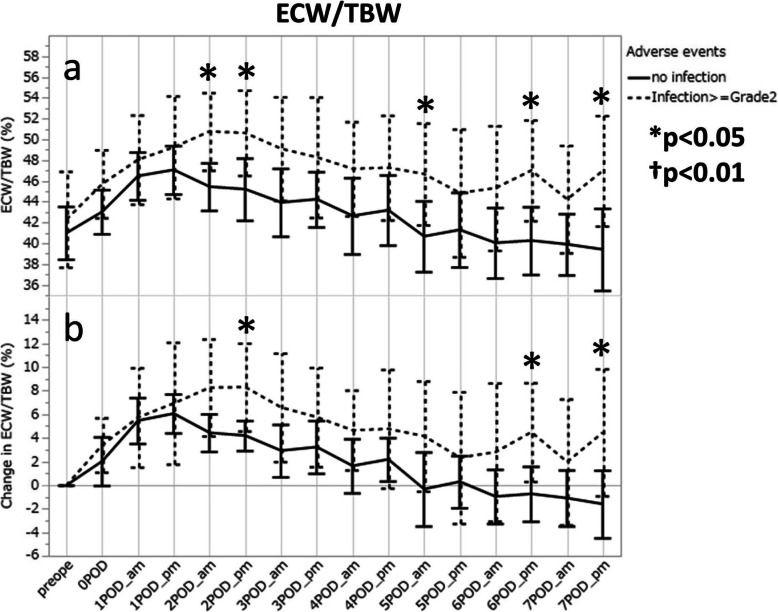
Changes in ECW/TBW after open esophagectomy in patients, with and without infectious complications; actual values (**a**) and increments (**b**). The mean values and the 95% confidential intervals are presented to show the trends within the same groups. ECW, extracellular water; TBW, total body water. *p < 0.05, †*p* = 0.01 by Mann-Whitney U test

The median value (25th percentile, 75th percentile) of ECW/TBW was higher in the infection group than in the non-infection group throughout the measurement period, and statistical significance was initially recognized in the morning of POD2 [50.2% (47.8, 55.3) vs 46.2% (41.9, 48.5), *p* = 0.0106] (Fig. 5a). It peaked in the evening of POD1 in the non-infection group, while the highest value was much greater in the infection group and appeared later on POD2 when ECW/TBW values already showed a decreasing trend in the non-infection group. The difference in ECW/TBW-C between the two groups was also significant in the evening of POD2 (Fig. 5b), but it was not as remarkable as the difference in the measured ECW/TBW values.

Linear regression analyses revealed that none of the factors listed on Table 2 affected the ECW/TBW values on POD2 at any time points.

### ROC analyses of BIA parameters, body weight changes, and CFBs regarding postoperative infection

As ECW-C, ICW/FFM and ECW/TBW values were observed to be significantly different according to the presence of infectious complications on POD2, ROC curve analyses were employed to evaluate the performance of each value for diagnosing infectious adverse events. The cut-off values and the values of the area under these ROC curves (AUROC) in the morning and evening are shown in Table 3. As the references, ROC curve analyses were also employed for the body weight changes and the CFBs in the same manner using the data on POD2. The AUROC of ICW/FFM in the morning of POD2 was the highest, at 0.93, with a cut-off value of 36.7%. That in the evening of POD2 was also high, at 0.83, with a cut-off value of 37.2%. The remaining AUROCs of ECW-C and ECW/TBW were equivalently high, employing the optimal cut-off value for each. All of these AUROC values were better than those calculated from the body weight changes and CFBs.

**Table 3:** ROC analysis in the prediction of infectious complications with each parameter on POD 2

Parameter	Timing	AUROC	Cut-off value	Sensitivity	Specificity
ECW change	2POD am	0.83	+ 3.0 kg	90.7%	66.7%
	2POD pm	0.84	+ 3.9 kg^a^	83.3%^a^	75.0%^a^
ICW/FFM	2POD am	0.93	36.7%	90.9%	100%
	2POD pm	0.83	37.2%	83.3%	83.3%
ECW/TBW	2POD am	0.81	47.8%	81.8%	75.0%
	2POD pm	0.80	49.0%	75.0%	83.3%
BW change	2POD	0.75	+ 2.5 kg	91.7%	66.7%
CFB/ preop.BW	2POD	0.72	+ 101.1 mL/kg	75.0%	66.7%

The cut-off values and the sensitivity and specificity values are shown with AUROC values

^a^Setting the cut-off value at + 4.2 kg also achieved a sensitivity of 75.0% and a specificity of 83.3%. All cut off values were defined by Youden index. *AUROC* Area under the Receiver Operating Characteristics curve, *ECW* extracellular water, *ICW* internal cellular water, *FFM* fat free mass, *TBW* total body water, *BW* body weight, *CFB* cumulative fluid balance, *preop* preoperative

### Multivariate logistic regression analyses of BIA parameters on POD2

Multivariate logistic analyses were also carried out to prove whether the measured BIA values on POD2 could be considered as the independent predictors of the morbidity. The measured BIA values on POD2 except for ICW/FFM in the morning were independent significant predictors for the infectious complications (all *p* < 0.01), while the patient factors (sex, age, preoperative body mass index (BMI), neoadjuvant therapy, ASA-PS, and preoperative PNI) and the surgical factors (lymph node dissection, reconstruction route, operative time, intraoperative blood loss, intra-operative RBC transfusion, and resection margin) were not statistically significant.

## Discussion

We herein evaluated the changes in water distribution in patients undergoing transthoracic esophagectomy. The ECW value rose overall for 2 days, decreasing thereafter and reaching the baseline 6–7 days after esophagectomy, while the ICW value remained stable throughout the postoperative course. This observation is highly consistent with the phenomenon known as “third spacing”, representing the fluid shift from the intravascular to the nonfunctional interstitial space. It implies a reduced blood volume and increased fluid in the interstitial area, i.e. tissue edema, and is essentially associated with reduced cardiac output and systemic hypoperfusion unless appropriate fluid replacement is given to maintain the patient’s blood pressure. Therefore, fluid resuscitation appears to be required for the initial 2 days and fluid restriction to prevent fluid overload in the subsequent postoperative days. This is the first report, to our knowledge, demonstrating the trends in BIA parameters measured for one week after transthoracic esophagectomy. The ECW-related values (ECW/FFM and ECW/TBW) peaked at the conventionally estimated onset of the fluid retention, or even earlier, in some patients. The BIA measurements appear to be useful for predicting and confirming fluid retention.

Transthoracic esophagectomy procedures encompass a broad range of surgical fields including the neck, thoracic and abdominal cavities. Moreover, direct compressions of the patient’s right lung or heart, and also one-lung ventilation can damage the cardiopulmonary system, especially during the transthoracic procedure. Transthoracic esophagectomy is clearly one of the most invasive surgeries. It is associated with enlargement of the interstitial fluid compartment, thereby necessitating very careful fluid management.

ECW-C was more than + 4 kg on POD2 on average, and the amount as well as the onset of the peak differed according to the presence of infectious complications. It is noteworthy that the ECW and ECW/TBW, which had increased, were restored to their baseline values on POD5 in patients without infectious complications, while remaining above baseline values until POD7 in those with infections. ECW values were generally very high in critically ill patients, especially those with sepsis [21]. ECW excess was not diminished in non-survivors with sepsis, in contrast to ECW which was significantly decreased from the baseline in surviving sepsis patients [22]. These observations might support our results, at least in part, indicating sustained increases in ECW over the baseline to be associated with sustained systemic inflammation followed by the development of infectious complications.

The ECW-C and ECW/TBW values differed significantly, especially on POD2, regardless of whether or not infectious complications developed. This might be attributable to infectious adverse events being associated with an increase in the interstitial fluid compartment, potentially leading to an increased demand for infusion therapy to maintain hemodynamics before the complications become clinically evident. This implies that postoperative infections might be the mechanism underlying abnormal excessive ECW storage before these adverse events manifest clinically during the postoperative course.

Fluid management strategies reportedly affect the incidence of postoperative adverse events. Fluid restriction and early-goal-directed therapy reportedly produce more favorable post-surgical outcomes than administering higher volumes of fluid without hemodynamic goals [4, 5]. A stratified meta-analysis showed a lower incidence of pneumonia and shorter ICU stays with goal-directed fluid management than with less strict fluid management protocols [5]. Stroke-volume guided goal-directed fluid management was also reported to reduce the occurrence of pneumonia, mediastinal abscesses and gastric tube necrosis, as well as shortening the ICU stay for post-esophagectomy patients [23]. We did not employ the “restricted” fluid management strategy and thus could not rule out the possibility of a causal relationship between excessive fluid infusion and infections in our prospective observational study, although we did not change the postoperative fluid management strategy for our patients. The CFB values between the infection and non-infection groups were significantly different only from POD 3 to 5, which appeared later than the significant difference in the ECW related parameters and also later than the occurrences of any infectious complications. The trends in BIA measurement changes according to the fluid management strategy and type of intravenous fluid (crystalloid or colloid) warrant further study.

ICW/FFM was the most predictive parameter with its high AUROC of 0.93 in the morning of POD2 when the cut-off value was set at 36.7%, although it was significantly lower in the infection group throughout almost the entire measurement period. One plausible explanation is that the excessive increase in the ECW compartment was significantly greater in the infection group. However, given that the ICW/FFM values were significantly lower in the infection group before and just after surgery, we can speculate that the baseline characteristics might have differed between the two groups. Studies have shown multi-frequency BIA to be useful for estimating muscle cross-sectional area [24] and strength as well. ICW amounts are known to correlate with the skeletal muscle mass, while ECW shows an inverse correlation. The ECW/ICW ratio in skeletal muscle is inversely correlated with gait speed independently of age, sex, body mass index and skeletal muscle mass [25], suggesting a positive relationship between low ICW amount and sarcopenia status. In addition, ICW shows an inverse correlation with frailty [26], such that a low ICW/FFM value at baseline might indicate sarcopenia and/or frailty affecting patients in the infection group. Studies have suggested sarcopenia to be a predictor of postoperative respiratory adverse events after esophagectomy [ 27, 28], observations which may support our aforementioned inferences.

Our results might reflect the mechanisms by which BIA parameters change, with or without infectious complications, after esophagectomy as shown in Additional file 2: Figure S2; secondary fluid excess in response to the infectious complications was confirmed by the significant increase in ECW, delayed peak and sustained increase in ECW in the presence of such complications. Furthermore, low preoperative ICW/FFM values before surgery exacerbated the increase in the ratio of ECW to TBW or FFM. It might be feasible to establish a better fluid management strategy based on the BIA parameters measured, serving as surveillance targets during the perioperative period. The multivariate analysis results in this study will also support the adequacy of this management strategy, although the BIA parameter values can be affected by the patient’s age, sex, and the status of neoadjuvant therapy and intraoperative RBC transfusion.

This study has limitations. First, the number of patients analyzed in each group was small, and statistical differences in BIA parameters might not reflect the actual effects of infectious complications. Second, patients with minimally invasive esophagectomy were not included in this study, and we therefore cannot identify differences in BIA measurements between the open and minimally invasive approaches. Finally, the patients were all Japanese and the cut-off ECW/TBW and ICW/FFM values for predicting infectious events may vary according to the ethnicity of the population.

## Conclusion

The peaks of ECW and ECW/TBW values differ according to the development of various infectious complications, and the ECW/TBW and ICW/FFM values on POD2 predict infectious adverse events after transthoracic esophagectomy. BIA measurements appear to be useful after transthoracic esophagectomy not only for assessing fluid retention and possibly allowing more meticulous fluid management, but also for predicting postoperative infectious adverse events based on the very high AUROC values obtained.

## Supplementary information

**Additional file 1: Figure S1.**Comparison of the times of the appearance of the peaks of each parameter with the postoperative fluid retention time estimated from the body weight change after surgery. The onset of fluid retention was defined as the first day of consecutive weight loss for two days after surgery. TBW, total body water; ECF, extracellular water; ICW, internal cellular water; FFM, fat-free mass.

**Additional file 2: Figure S2.** Summary of the body water distribution in patients after transthoracic esophagectomy. The amount of ECW increases after surgery and peaks at approximately Day1–18:00 without infection, but when an infection is present the peak is seen later, around Day2–18:00, and the ECW volume also becomes even higher. Patients with low preoperative ICW/FFM might be more dramatically affected in terms of their ICW/FFM and ECW/TBW percentage by this ECW volume change, which may result in higher sensitivity of these parameters as predictors of infectious complications after esophagectomy. ECW, extracellular water; ICW, internal cellular water; FFM, fat free mass; TBW, total body water.

## Data Availability

The datasets used and/or analyzed during the current study are available from the corresponding author on reasonable request.

## Abbreviations

Af: Atrial fibrillation
ARDS: Acute respiratory distress syndrome
ASA-PS: Physical state classification by the American Society of Anesthesiologists
AUROC: Area under receiver operating characteristic curve
BIA: Bioelectrcial impedance analysis
BMI: Body mass index
BW: Body weight
C-D classification: Clavien-Dindo classification
CFB: Cumulative fluid balance
CRP: C-related protein
CT: Computerized tomographic scanning
ECW: Extracellular water
ECW/FFM-C: Change in ECW/FFM
ECW/TBW-C: Change in ECW/TBW
ECW-C: Change in ECW
FFM: Fat-free mass
ICW: Internal cellular water
ICW/FFM-C: Change in ICW/FFM
ICW-C: Change in ICW
Laryngeal N: Laryngeal nerve
PNI: Prognostic nutritional index
POD: Postoperative day
pT/N/M: Pathological T/N/M
RBC: Red blood cell
ROC: Receiver operating characteristic
SSI: Surgical site infection
TBW: Total body water
TBW/FFM-C: Change in TBW/FFM
TBW-C: Change in TBW
UICC: The Union for International Cancer Control
WBC: White blood cell
