# Supplementary figures and images for: Perioperative fluid dynamics evaluated by bioelectrical impedance analysis predict infectious surgical complications after esophagectomy
Source: BMC Surg. 2019 Dec 2;19:184. doi: 10.1186/s12893-019-0652-z (PMC6889694; doi:10.1186/s12893-019-0652-z)

## Slide 1
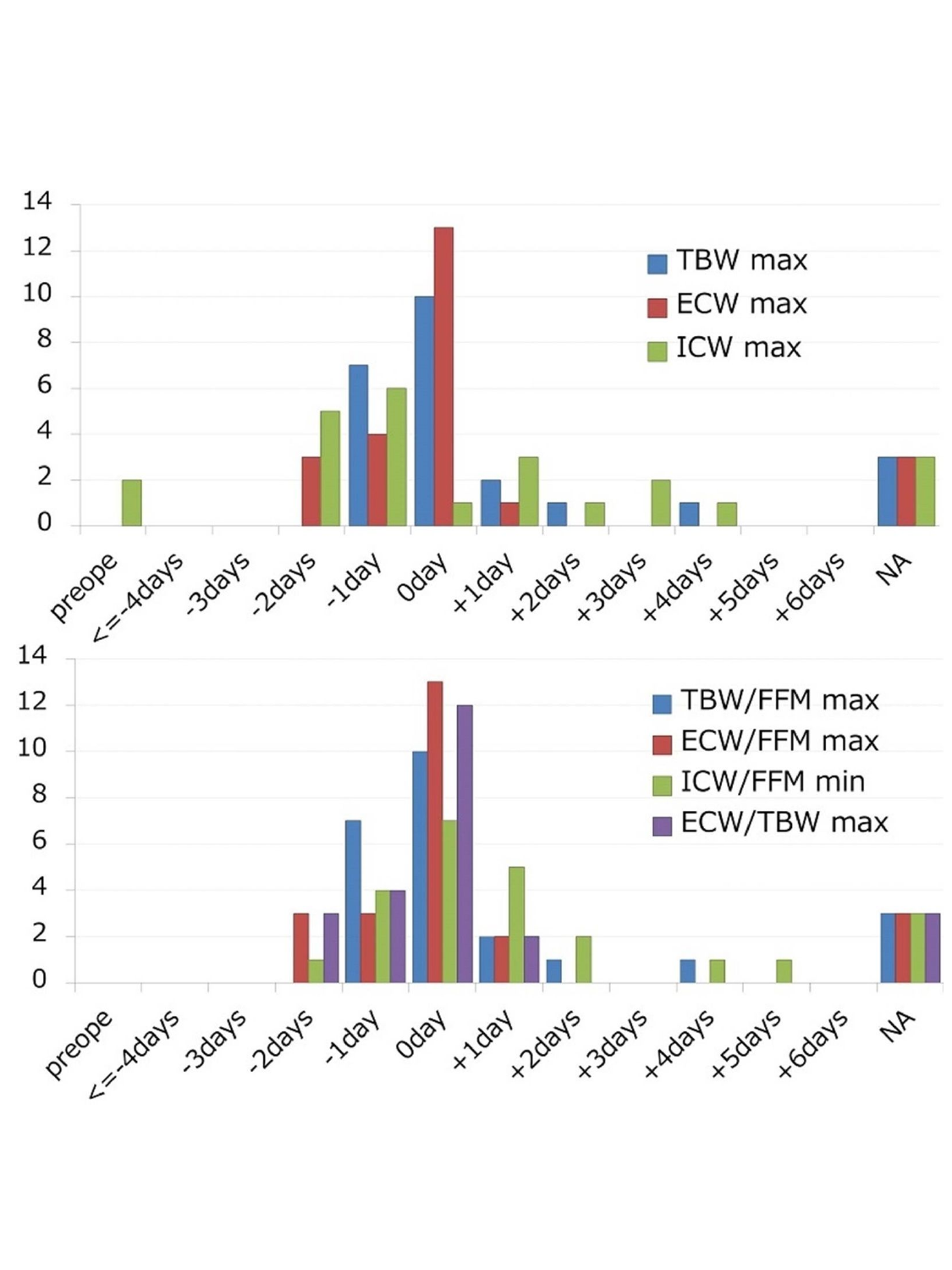

Supplement: Supplementary file 1 — Additional file 1: Figure S1.Comparison of the times of the appearance of the peaks of each parameter with the postoperative fluid retention time estimated from the body weight change after surgery. The onset of fluid retention was defined as the first day of consecutive weight loss for two days after surgery. TBW, total body water; ECF, extracellular water; ICW, internal cellular water; FFM, fat-free mass. [file 12893_2019_652_MOESM1_ESM.pptx]

## Slide 1
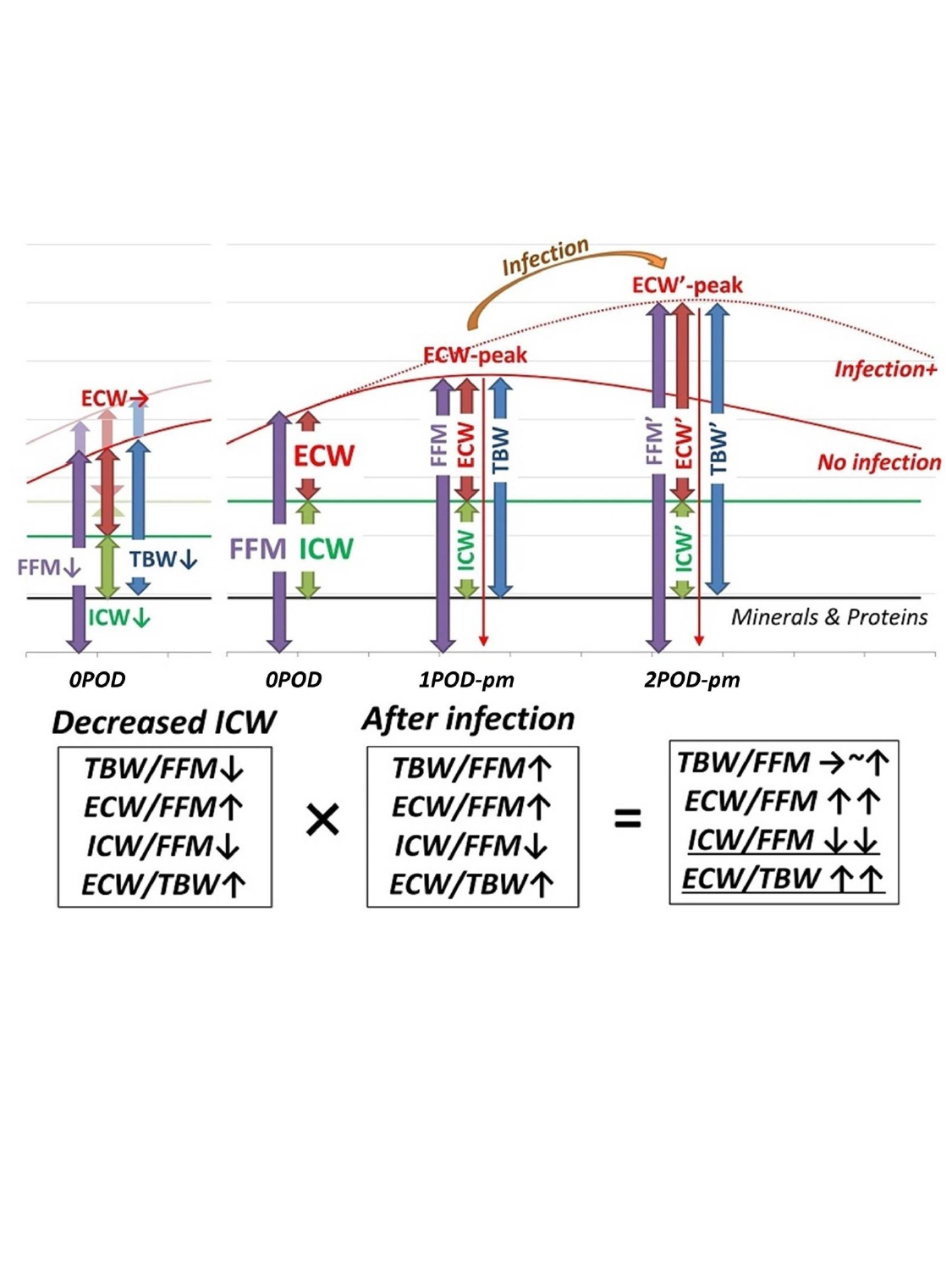

Supplement: Supplementary file 2 — Additional file 2: Figure S2. Summary of the body water distribution in patients after transthoracic esophagectomy. The amount of ECW increases after surgery and peaks at approximately Day1–18:00 without infection, but when an infection is present the peak is seen later, around Day2–18:00, and the ECW volume also becomes even higher. Patients with low preoperative ICW/FFM might be more dramatically affected in terms of their ICW/FFM and ECW/TBW percentage by this ECW volume change, which may result in higher sensitivity of these parameters as predictors of infectious complications after esophagectomy. ECW, extracellular water; ICW, internal cellular water; FFM, fat free mass; TBW, total body water. [file 12893_2019_652_MOESM2_ESM.pptx]
